# Identification of CβS and ODC antimony resistance markers in anthroponotic cutaneous leishmaniasis field isolates by gene expression profiling

**DOI:** 10.1016/j.parepi.2025.e00413

**Published:** 2025-01-23

**Authors:** Farzaneh Zarrinkar, Iraj Sharifi, Razieh Tavakoli Oliaee, Ali Afgar, Elaheh Molaakbari, Mehdi Bamorovat, Zahra Babaei, Ebrahim Eskandari, Ehsan Salarkia, Marzieh Asadi

**Affiliations:** a Leishmaniasis Research Center, Kerman University of Medical Sciences, Kerman, Iran; b Center for Hydatid Disease, Kerman University of Medical Sciences, Kerman, Iran; c Center for Research and Training in Skin Diseases and Leprosy, Tehran University of Medical Sciences, Tehran, Iran; d Toxoplasmosis Research Center, Department of Parasitology, School of Medicine, Mazandaran University of Medical Sciences, Sari, Iran

**Keywords:** Antimonial resistance, *Leishmania tropica*, Genetic markers, Cystathionine β synthase, Ornithine decarboxylase

## Abstract

Antiparasitic resistance represents a serious global public health concern with tremendous economic and safety implications. This study intended to investigate the expression of the two major resistant markers: cystathionine β synthase (CβS) and ornithine decarboxylase (ODC) in antimony unresponsive *Leishmania tropica* isolates compared to responsive ones. Twenty-six patients were randomly selected from widely known foci of anthroponotic cutaneous leishmaniasis in southeastern Iran. Written informed consent of the patients was obtained. Two smears were prepared from the edge of each active lesion; one for microscopic direct smear preparation and the other for inoculation into monophasic NNN media, then for mass production of promastigotes into RPMI-1640 monophasic culture for performing nested PCR and gene expression quantification by real-time PCR. Twenty-six patients consisting of 13 unresponsive and 13 responsive equally distributed among female and male groups. All cases were identified to be *L. tropica.* Both resistant gene markers were significantly up-regulated in unresponsive and responsive isolates. The findings showed that CβS and ODC are directly linked with the resistance to L. *tropica.* Alternative drugs or combination therapy and monitoring drug resistance to prevent the spread of resistant isolates are proper strategies to control the disease.

## 1 Introduction

Leishmaniasis inflicts significant physical, psychological, and socioeconomic harm on millions of people worldwide, mostly in tropical and subtropical areas. This complex disease debilitates, disfigures, and can be fatal if not treated ("World Health Organization, 2023). Leishmaniasis causes immense human suffering. It affects some of the world's most vulnerable people, who often live in remote and inaccessible communities, thereby along with other neglected tropical diseases (NTDs) creating a vicious cycle of poverty in vulnerable and impoverished countries. Leishmaniasis affects nearly one billion people, afflicts 1.3 million, and kills about 30,000 each year (*Leishmaniasis, World Health Organization, Key Facts*, 2023). The disease presents as a self-healing local cutaneous lesion (CL), mutilating type mucocutaneous (MCL) to fatal systemic leishmaniasis (VL), although other diverse clinical presentations depending on the causative species, geographical location, and immune response of the host are produced (Mathison and Bradley, 2023).

In the absence of an approved vaccine, leishmaniasis treatment relies on chemotherapy. Meglumine antimoniate (Glucantime®), and also sodium stibogluconate (Pentostam®) formulations known as pentavalent antimonial (SbV), have been the primary drugs for treating leishmaniasis in the past eight decades (de Almeida et al., 2024). However, the effectiveness of SbV is declining, leading to a 65 % treatment failure rate in Bihar, India (Chhajed et al., 2023). Resistance to SbV has been observed in various regions, including Asia (Yajima et al., 2023; Bamorovat et al., 2018a), North Africa (Blaizot et al., 2024), Latin America (Navarro and Visbal, 2024), and the Indian Subcontinent (van Griensven et al., 2024) While some treatment failures may be due to factors such as the host's immune response and pharmacological properties (Bamorovat et al., 2019). The primary reason for the decrease in SbV efficacy is the parasite's development of drug resistance (Bamorovat et al., 2021). Given the limited alternatives for treating leishmaniasis, there is an urgent need to elucidate the role of SbV resistance markers in *Leishmania* parasites. Knowledge of parasite different responses to resistance indicators helps design proper control strategies to manage the disease.

Evidence suggests that resistance to antimony is a complex process involving genetic, metabolic, and lipid composition changes. Parasites resistant to antimony have demonstrated various strategies to counteract the drug's effects, including reducing drug uptake by decreasing the aquaglyceroporin 1 transporter, enhancing antioxidant responses through thiol metabolism activation, adjusting energy metabolism to support the increased energy demands of the antioxidant response, utilizing thiols as drug binders to create thiol-metal complexes, and boosting the efflux of thiol-metal complexes through exocytosis by overexpressing ABC transporters (Oliaee et al., 2018). However, the specific molecular mechanisms that coordinate these responses to combat antimony are not yet understood. It is essential to unravel these processes to enhance the development of new therapeutic approaches, such as combination therapies to restore the effectiveness of SbV, and to delve deeper into the fundamental biology of the *Leishmania* parasite. Parasite resistance to SbV is increasing in ACL leishmaniasis caused by L. *tropica*, which can be due to the decrease in sensitivity to SbV, insufficient treatment period, and the person-to-person (anthroponotic) transmission cycle, which is the only measure for controlling the disease (Aflatoonian et al., 2019).

Iran is among the 10 countries that have the highest number of estimated cases (Alvar et al., 2012). The main focus of Acl in the southeast of Iran is Kerman province, where more than 97 % of autochthonous cases are caused by *L. tropica* parasite (Sharifi et al., 2015). Various risk factors have been identified in creating unresponsive isolate (Bamorovat et al., 2018b; Aflatoonian et al., 2016; Aflatoonian et al., 2013). Among the patients with ACL in well-known endemic foci, 10.8 %, 11 %, and 11.35 % did not respond to treatment with Glucantime® in the cities of Mashhad (Hadighi et al., 2006), Bam (Pour et al., 2011), and Kerman (Karvar et al., 2016), respectively. It has been determined that SbV must be converted to trivalent active form to have a leishmanicidal effect on the promastigote and amastigote of the parasite (Shaked-Mishan et al., 2001). The report of treatment failure and resistance to SbV compounds from 25 years ago, the report includes. Brazil (Oliveira-Neto et al., 1997), Bolivia (Bermudez et al., 2006), Colombia (Palacios et al., 2001), India, Iran (Sarkari et al., 2016) and Peru. The most extreme situation takes place in Bihar (India) where 50–65 % of the patients are unresponsive to treatment (Sundar, 2001).

During this digentic life, the parasite is affected by various oxidative or nitrosative stresses caused by reactive oxygen species (ROS) or reactive nitrogen species (RNS). They are produced to prevent the immune system of the host (Dostálová and Volf, 2012; Van Assche et al., 2011). SbV compounds such as Sodium Stibgluconate or Meglumine Antimoniate are still the drug of choice in the first line of human leishmaniasis treatment (Frezard et al., 2013). The pentavalent form of Sb (Sbv) stimulates macrophages to produce ROS and nitric oxide (NO), which indirectly induces oxidative and nitrosative stress on the parasite. The production of ROS and NO in infected macrophages is stimulated by the activation of PI3K or MAPK (Mookerjee Basu et al., 2006). Also, the reduced form of the drug SbIII connects to the thiols of the parasite and prevents the effective action of trypanothione causes oxidative/nitrosative stress, and causes the death of the parasite by disrupting the redox balance (Baiocco et al., 2009). What is certain is that parasites are exposed to oxidative stress (ROS) or nitrosative stress (RNS) during their life cycle. To deal with these stresses, parasites use a strong defense system that consists of molecules and enzymes that destroy this reactive species (Krauth-Siegel and Comini, 2008).

This defense system is based on low molecular weight thiols such as glutathione and trypanothione, which play an essential role in the antioxidant defense of parasites by regulating redox homeostasis (Fairlamb et al., 1985). The synthesis of glutathione and trypanothione depends on the sulfur-containing amino acid, cysteine, an amino acid that plays an important role in the biological process of protein stability, structure, regulation of catalytic activity, and post-translational modifications (Valashani et al., 2024). Cysteine  biosynthesis is done by *de novo* or reverse transsulfuration (RTS) routes. In the RTS pathway, it leads to the production of cysteine from methionine through the formation of a cystathionine intermediate (Thomas and Surdin-Kerjan, 1997). These reactions are catalyzed by two enzymes. First, cystathionine β synthase (CβS), which synthesizes cystathionine from homocysteine  and serine, and second, cystathionine γ-lays, which produces cysteine  from cystathionine (Aitken and Kirsch, 2005).

**Fig. 1:**
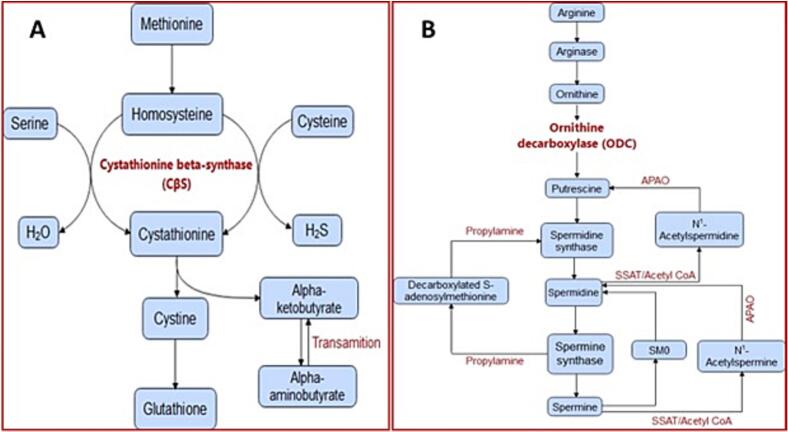
There is an association between polyamine levels and drug resistance, where higher levels of polyamines contribute to increased resistance to drugs. *Leishmania's* defense system is based on low molecular weight thiols such as glutathione, ornithine, and trypanothione, which play an important role in the antioxidant defense of parasites by regulating redox homeostasis. These reactions are catalyzed by two enzymes (cystathionine β synthase (CβS). Polyamines are essential for the growth, infectivity, and survival of the parasite living in mammalian hosts.

Considering the importance of thiol metabolism in *Leishmania* species, these parasites depend on thiol metabolism during their digenetic life cycle to respond to oxidative/nitrosative stress. Considering that L-cysteine  plays an essential role in the synthesis of thiol-related antioxidant molecules. In the present study, the role of the gene encoding the key enzyme of the cysteine  biosynthesis pathway (CβS) against SbV compounds is determined. On the other hand, the conversion of L-ornithine into putrescine by the enzyme CβS is followed by its transformation into spermidine by spermidine synthase (Fig. 1A) (Müller et al., 2001). In *Leishmania* parasites, polyamines such as spermidine contribute to their growth and resistance to AsIII/antimony (Haimeur et al., 1999; Singh et al., 2007; Birkholtz et al., 2011) Increased levels of Ornithine decarboxylase (ODC) have been observed in SbV-resistant L. *donovani* field isolates and L. *tarentolae* mutants resistant to SbV and AsIII respectively (Fig. 1B) (Haimeur et al., 1999; Mukherjee et al., 2007; Rai et al., 2013). However, the exact role of ODC and CβS in SbIII resistance with ACL with L. *tropica* has not been fully studied yet. No data are available to describe the role of CβS and ODC in responsive and unresponsive cases with CL. In this study, the expression of CβS and ODC as resistance genes in isolates from patients with ACL due to L. *tropica* is investigated.

## 2 Materials and methods

### 2.1 Ethical statement

Ethical approval was granted by the Ethical Committee of Kerman University of Medical Sciences protocol no: 98000753 and ethics no: IR.KMU.REC.1398.516, and that written informed consent of the patients was also obtained. Unresponsive patients were referred to the Educational University Hospitals for further checkups and laboratory examinations for combination treatment, while demographic data were kept confidential.

### 2.2 Study area

In the southeastern province of Kerman, there are two clinics, one in Bam and the other in Kerman district, that have been serving as referral centers for the treatment of patients with CL since 2005. These clinics offer clinical examination, detection of cases, diagnosis, and treatment using meglumine antimoniate alone or in combination. The clinics have well-trained health officers and experienced physicians who provide services free of cost to patients. The national CL protocol, following WHO guidelines, was first implemented in these clinics after the CL epidemic in Bam (Karvar et al., 2016).

### 2.3 Clinical isolates

**Fig. 2:**
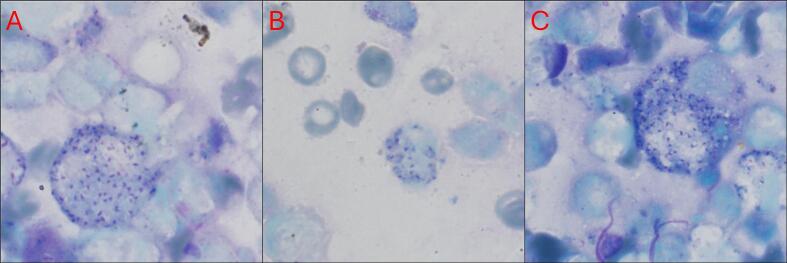
Representative images of positive direct microscopic samples of cutaneous leishmaniasis cases due to *Leishmania tropica* (A, B, and C).

Clinical isolates were obtained from patients who were referred to health centers between February 2021 and January 2022. The samples were collected from 26 patients, with 13 being unresponsive and 13 responsive to Glucantime® treatment (Sanofi-Aventis, Paris, France). Both groups were subjected to positive direct microscopic examination (Fig. 2).

### 2.4 Unresponsive and responsive cases

**Fig. 3:**
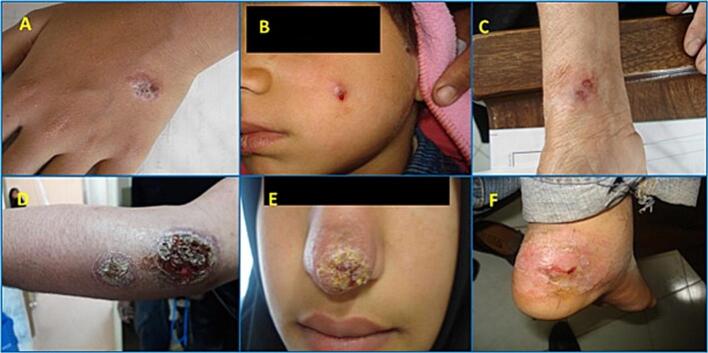
Representative images of patients with cutaneous leishmaniasis lesions caused by *Leishmania tropica* obtained from endemic areas of Kerman province. Responsive cases: A, B, and C. Unresponsive cases: D, E, and F*.*

Unresponsive cases are patients who are still showing active lesions after receiving two rounds of Glucantime® treatment *via* systemic (20 mg/kg/day for 3 weeks) or intralesionally administration (once a week for 12 weeks coupled with cryotherapy by biweekly administration of liquid nitrogen). On the other hand, patients who were completely cured after a single round of Glucantime® treatment with or without cryotherapy as mentioned above, and did not have any relapses during six months of follow-up examinations with complete re-epithelialization of the lesion, were considered responsive cases (Fig. 3).

### 2.5 Sampling and parasite culture

Skin samples were taken from the margin of the lesion using a scalpel and blade (no. 15) and isolated with an NNN medium. The isolated parasites were subcultured in RPMI-1640 medium (Biosera, France) supplemented with 10 % heat-inactivated (56 °C for 30 min) fetal bovine serum (Gibco, Germany), penicillin (100 IU/ml), streptomycin (100 μg/ml) and incubated at 25 ± 1 °C in 25 cm^2^ culture flasks. *L. tropica* strain MHOM/IR/10/175 (S175) and MHOM/IR/10/439 (R439) (7), which were used as antimony-sensitive and resistant reference strains, respectively, were recovered from liquid nitrogen and sub-cultured in complete RPMI-1640 medium. Harvested promastigotes were centrifuged (900 ×*g* for 10 min at room temperature) and washed with RPMI-1640 and pellets were stored at −70 °C for further molecular experiments.

### 2.6 Identification of Leishmania isolates

The genomic DNA of all cultured samples was extracted using the QIAamp DNA Mini Kit (Qiagen, Germany) according to the manufacturer's instructions. Molecular detection of *Leishmania* isolates was performed by nested PCR, which can amplify a variable region of the kinetoplast mini circle DNA (kDNA) for identification of *Leishmania* species. Nested PCR was carried out in two consecutive steps as described elsewhere (Aflatoonian et al., 2014).

In brief, two general primers of CSB2XF (CGAGTAGCAGAAACTCCCGTTCA) and CSB1XR (ATTTTTCGCGATTTTCGCAGAACG) were used as external primers, then in the second round, 13Z (ACTGGGGGTTGGTGTAA AATAG) and LiR (TCGCAGAACGCCCCT) were used as specific internal primers. The final PCR products were separated on 1.5 % agarose gel electrophoresis and

visualized under UV Transilluminator (Uvitech, Cambridge, UK).

### 2.7 Gene expression quantification

The entire RNA of the early stationary phase promastigotes (5 × 10^7^/ml) of 26 unresponsive and responsive isolates was extracted using the High Pure RNA Isolation Kit (Roche, Basel, Switzerland). Initially, the concentration of RNA was determined using Thermo Fisher Scientific nanodrop, and then cDNA was synthesized by Roche Synthesis Kit. The remaining qRT-PCR processes were carried out based on the protocol reported elsewhere. In Table 1, the primer's pattern and control gene sequences are presented. The relative expression of resistance genes in L. *tropica* field isolates was determined based on the cycle threshold (Ct) value of the target gene with that of the reference gene, 40S ribosomal protein, to normalize the results relative to the resistant and sensitive reference isolates. Differential expression of a clinical sample compared to the sensitive reference isolate of S175 was expressed as the fold change. The following equation was used to calculate CT: [ΔCT = CT (target) - CT] (Livak and Schmittgen, 2001). Moreover, the fold change was considered using the comparative threshold approach (ΔΔCT).

**Table 1:** The specific primers and reference gene sequences.

Templet	Forward and reverse sequences (5´-3´)	Product size (bp)
CBS	F- GCACGGAGAACGTCTCTTCC R-CACACTGGTCGTAGATCTCCTG	162
ODC	F-TGGTGCGCCCTTACTTTGC R-TTGCACGGGTTGGCGAAG	169
40s	F-GTTGAGGTGCGTGGTCTGTC R- TGCAGGTTGCTCAGGAGCTT	71

### 2.8 Statistical analysis

Data were analyzed using an independent sample *t*-test. Also, Pearson's rank correlation coefficients were used. *P*-values for significant differences in the expression levels of unresponsive and responsive parasites of different genes were calculated with GraphPad Prism 7.01 (GraphPad Software, Inc., San Diego, CA, USA). The results are presented as the mean standard deviations (SDs). *P* < 0.05 was considered to be statistically significant.

## 3 Results

### 3.1 Clinical data

**Fig. 4:**
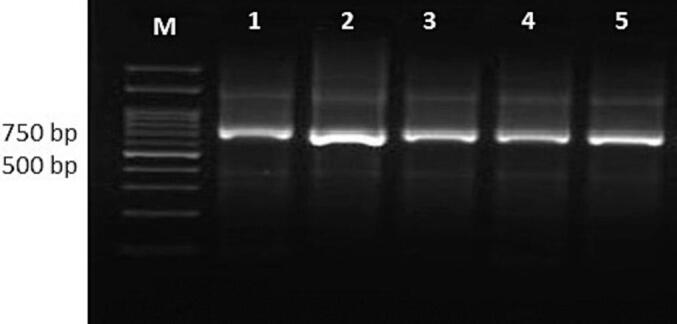
Identification of clinical isolates and two reference strains using nested PCR. Lanes 1–3, *Leishmania tropica* isolates; lane 4, sensitive L. *tropica* reference strain; lane 5, resistant L. *tropica* reference strain; lane M, 100 bp DNA size marker.

The study recruited 26 patients with ACL lesions who live in areas where CL disease is endemic in Kerman Province, Southeast Iran. They were categorized as either unresponsive or responsive patients (Table 2). Diagnosis of *L.tropica* was confirmed through three methods: positive direct smear preparations, the culture of promastigotes in an NNN isolation medium, and nested PCR. The entire field isolates and both reference strains displayed the specific band of 750 bp with nested PCR; *L. tropica* was the only species identified as the causative agent in all our CL patients (Fig. 4).

**Table 2:** Baseline demographic and clinical characteristics of unresponsive patients with anthroponotic cutaneous leishmaniasis caused by *Leishmania tropica.*

Demographic characteristics			Lesion characteristics				Diagnosis		
No.	Sex	Age (year)	No	Size (cm)	Location	Type	Culture	Nested-PCR	Duration of illness (month)
1	Male	9	1	2 × 1	Face	Ulcerated	Positive	*L. tropica*	37
2	Female	24	1	2.2 × 1	Arm	Nodular	Positive	*L. tropica*	11
3	Female	8	1	3 × 1	Lower Lip	Ulcerated	Positive	*L. tropica*	24
4	Male	8	1	5 × 1.7	Arm	Ulceronodular	Positive	*L. tropica*	17
5	Male	10	1	2 × 2	Face	Nodular	Positive	*L. tropica*	12
6	Male	5	1	3.1 × 1.5	Lower Lip	Ulcerated	Positive	*L. tropica*	18
7	Female	16	1	0.5 × 1	Leg	Nodular	Positive	*L. tropica*	12
8	Male	8	1	0.5 × 1.4	Upper Lip	Nodular	Positive	*L. tropica*	12
9	female	7	1	3 × 3	Arm	Ulcerated	Positive	*L. tropica*	12
10	Male	11	1	3 × 2	Face	Ulcerated	Positive	*L. tropica*	24
11	Female	48	1	2 × 1	Hands	Nodular	Positive	*L. tropica*	6
12	Male	57	1	4 × 2	Arm	Ulceronodular	Positive	*L. tropica*	9
13	Male	22	1	2 × 1	Upper Lip	Nodular	Positive	*L. tropica*	12

Five of the unresponsive patients were female while eight were male, and their ages ranged from 5 to 57 with a mean ± SD age of 17.9 ± 16.5 years. For male and female groups, the mean ± SD age was found 16.2 ± 17.2 and 20.6 ± 16.8 respectively. The responsive group consisted of six females and seven males, with a mean ± SD age of 23.1 ± 12.8 years old. The study found that ulcerated and nodular lesions were the most common clinical forms in both responsive and unresponsive patients, making up 38 % and 46 % of observed lesions respectively. Unresponsive patients had more lesions on the face (54 % on the cheek and around the lips), arms and hands (38 %), and one on the leg. In responsive patients, 71.5 % of the lesions were observed on the hands and arms. The mean ± SD size of unresponsive and responsive lesions was 2.5 ± 1.2 and 1.8 ± 0.7 cm, which ranged from 0.5 to 5 and 0.5 to 1.8 cm, respectively (Table 2).

### 3.2 Gene expression analysis of L. *tropica* clinical isolates

**Fig. 5:**
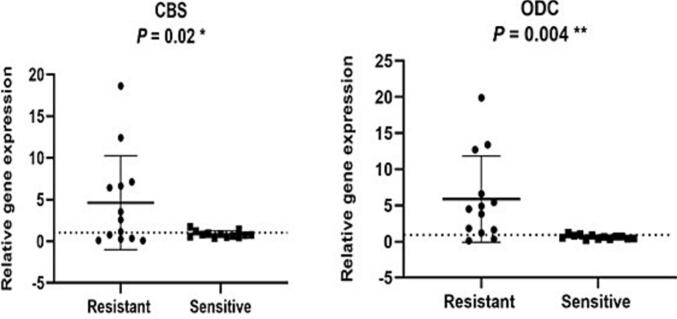
Expression level of 2 target genes in *Leishmania tropica* isolates from unresponsive and responsive patients by real-time qPCR. The expression of 40S was used to normalize the data. The values are the mean SD of three independent experiments (**P < 0.01 and *P < 0.05).

The gene expression levels of two target genes in 26 clinical isolates of L. *tropica* were measured by real-time qPCR relative to reference isolates S175 and R439 (Fig. 5). The isolates were divided into 13 unresponsive and 13 responsive groups. The results indicated that the CBS gene was upregulated in all unresponsive isolates by a fold of 1.5 to 18.2, with a significance level of 0.02. Similarly, the ODC gene was upregulated in all unresponsive isolates from 1-fold to 20-fold, with a significance level of 0.04.

## 4 Discussion

Antiparasitic resistance represents a serious global public health concern with tremendous economic and security implications. One of the main drivers of drug resistance is the excessive or inappropriate use of antiparasitics in people (Oliaee et al., 2018). Chemotherapy is the main approach to control leishmaniasis disease. One of the common treatments is the use of SbV, but they have many harmful side effects and a high rate of resistance (Sundar, 2001; Sudhandiran and Shaha, 2003; Croft et al., 2006; Croft and Olliaro, 2011; Andrade et al., 2016; Haimeur et al., 2000) To prevent or reverse resistance, it is essential to understand the molecular mechanisms responsible for it in *Leishmania* species, as this knowledge can help identify potential drug targets.

The identification of suitable markers that indicate resistance to drugs in patients with ACL can be beneficial in developing new strategies to maintain the effectiveness of currently used drugs. However, the resistance mechanisms found in laboratory and field isolates may differ (Kumar et al., 2012). Variability in the clinical response to SbV such as sodium stibogluconate and meglumine antimonate has been a persistent challenge in the treatment of leishmaniasis over the past decades. One possible explanation for this issue is the inherent differences in species' sensitivity to these medications. Research utilizing the amastigote-macrophage model has indicated that L. *donovani* and *L. brasiliensis* are approximately three to five times more responsive to sodium stibogluconate compared to *L. major, L. tropica,* and *L. mexicana* (Croft, 2001). A controlled clinical trial conducted in Guatemala further demonstrated that sodium stibogluconate led to a notably higher cure rate in patients with *L. braziliensis* lesions (96 %) compared to those with L. *mexicana* lesions (57 %). The report suggests that ulcerated and nodular lesions are frequently observed in patients with unresponsive and responsive responses and that the face and hands are the most commonly affected areas. These findings are consistent with previous research that has indicated papules and hands as the most common form and site of involvement in patients with ACL (Oliaee et al., 2018; Khosravi et al., 2017).

The information on the resistance of field isolates of *Leishmania* to antimonial, specifically L. *tropica*, is limited. In Mashhad City, 10.8 % of patients with ACL did not respond to Glucantime® treatment in the macrophage cultivation model. However, there was no observed difference between sensitive and resistant strains in terms of sequencing of the pteridine reductase 1 gene (Hadighi et al., 2006).

In a research study, scientists investigated the expression of a gene called activated protein kinase C receptor (LACK) in resistant and sensitive clinical L. *tropica* isolates. LACK is involved in important cellular functions like signaling pathways. The results showed that in sensitive isolates, the expression of LACK was increased, but it was not significantly decreased in resistant isolates (Hajjaran et al., 2016).

In the present study, the expression pattern of two important resistance gene markers including CβS and ODC in different clinical isolates was assessed. The data in our research indicate that the increased resistance to antimonial compounds, which are influenced by certain enzymes, could be explained by the higher levels of cysteine production. This is necessary to synthesize glutathione and trypanothione, which are required to detoxify antimony (Wyllie and Fairlamb, 2006; Wyllie et al., 2004).

Thiol is mainly present in Kinetoplastida but can be found in some other parasitic protozoa such as *Entamoeba histolytica* (Ondarza et al., 2005). This molecule is essential for the survival of these parasites, but it is absent from humans. Therefore, the enzymes that produce and utilize this molecule are potential targets for the development of new drugs to treat these diseases (Schmidt and Krauth-Siegel, 2002).

The increase of thiol levels has been identified as a major way that resistant *Leishmania* lines detoxify SbIII (Mukhopadhyay et al., 1996). In L. *amazonensis* promastigotes resistant to SbIII in the laboratory, cysteine, glutathione, and trypanothione levels were all increased significantly. However, only cysteine levels were significantly increased in SbIII-resistant *Leishmania infantum* parasites (El Fadili et al., 2005; do Monte-Neto et al., 2011). The levels of the thiols glutathione and trypanothione were found to be lower in clinical L. *donovani* isolates that are sensitive to SbIII treatment compared to those that are resistant (Mandal et al., 2007). In addition, antimony-resistant isolates showed increased expression of genes related to the biosynthesis of cysteine, glutathione, and trypanothione. These genes include CβS, γ-glutamyl cysteine synthetase, ornithine decarboxylase, trypanothione synthetase, trypanothione reductase, and spermidine synthase (Mukherjee et al., 2007; do Monte-Neto et al., 2011; Decuypere et al., 2012). In our results, the expression of CβS in unresponsive isolates showed an upregulated with a significance level (*P* = 0.02). The presence of cysteine in all thiols is important for *Leishmania tropica* to survive under oxidative stress. This raises the question of whether this is also true for other *Leishmania* species. Interestingly the effectiveness of antimony may be linked to changes in the levels of expression of two proteins, ODC and CβS. This suggests that cysteine, present in all thiols, may play a role in developing resistance to antimony. Thus, understanding the role of ODC and CβS enzymes in antimony resistance in *Leishmania tropica* can help identify new targets and design appropriate combination therapies for disease treatment.

Trypanosomatids have a unique redox system that relies on a low molecular mass thiol called trypanothione. This thiol is not present in mammals but is necessary for the survival of the parasites, making it a potential target for chemotherapy. Antimony is a compound that affects the parasite's intracellular redox balance by depleting trypanothione and glutathione and inhibiting trypanothione reductase (Wyllie et al., 2004). The overexpression of enzymes from the biosynthetic pathway of trypanothione, such as ODC and GSH1, has been linked to high trypanothione levels and antimony-resistant *Leishmania spp.* (Mukhopadhyay et al., 1996; Mandal et al., 2007). *Leishmania* can both create polyamines on *de novo* and obtain them from their host. The enzyme ornithine decarboxylase is crucial for the production of spermidine and is important for the parasite's growth and virulence. A strain of L. *donovani* knockout for this enzyme struggled to grow efficiently in both mice and macrophages until polyamine levels were restored through genetic or metabolic means (Boitz et al., 2009).

The importance of ODC for the survival of parasites *in vitro* and *in vivo* was supported by research that used DFMO, a specific inhibitor of the enzyme (Bacchi et al., 1980; Kaur et al., 1986). Several studies have found a correlation between polyamine levels and drug resistance, where higher levels of polyamines led to increased resistance to drugs. For example, the transfection of ODC in L. *donovani* led to increased levels of ODC, which produced parasites that were resistant to antimony (Singh et al., 2007). *In vitro* selected AsIII-resistant L. *tarentolae* showed increased levels of ODC mRNA, enzymatic activity, putrescine, and spermidine (Haimeur et al., 1999). However, there was no difference in ODC mRNA levels between SbIII-resistant and the parental cell line (Haimeur et al., 2000). Additionally, *L. donovani* field isolates that were resistant to sodium antimony gluconate (SAG) had an increased content and activity of ODC and polyamines, along with gene amplification, as well as of putrescine and spermidine (Singh et al., 2007) (Singh et al., 2007; Mukherjee et al., 2007; Rai et al., 2013). In our results, the expression of ODC in unresponsive isolates showed an upregulated with a significance level (*P* = 0.04).

This study shows that ODC and CβS were directly associated with the resistance of L. *tropica* to SbIII. The study suggests that a combination therapy using inhibitors of polyamine and/or glutathione/trypanothione biosynthesis could be an effective approach to reducing drug resistance, and toxicity and increasing the effectiveness of SbIII treatment for the disease caused by this parasite species.

To address drug resistance and treatment failure, alternative therapies such as miltefosine, amphotericin B, paromomycin, or combination therapies have been used. Monitoring drug efficacy, conducting drug susceptibility testing, and implementing proper treatment protocols are crucial in managing leishmaniasis and preventing the spread of drug-resistant strains. Drug resistance to antimonial drugs, particularly sodium stibogluconate and meglumine antimonate, has become a significant concern in the treatment of leishmaniasis. Resistance mechanisms can involve alterations in drug uptake, efflux pumps that expel the drug from the parasite, or changes in drug targets that reduce the drug's efficacy. Studies have shown that prolonged or repeated exposure to antimonial can lead to the development of resistance in *Leishmania* parasites.

## Funding

This project was supported by the 10.13039/501100004621Kerman University of Medical Sciences, Kerman, Iran (project no. 98000753).

## CRediT authorship contribution statement

**Farzaneh Zarrinkar:** Writing – review & editing, Writing – original draft, Visualization, Validation, Software, Methodology, Investigation, Formal analysis, Data curation. **Iraj Sharifi:** Writing – review & editing, Writing – original draft, Visualization, Validation, Supervision, Resources, Project administration, Methodology, Investigation, Funding acquisition, Conceptualization. **Razieh Tavakoli Oliaee:** Writing – review & editing, Writing – original draft, Software, Methodology, Formal analysis, Data curation. **Ali Afgar:** Writing – review & editing, Writing – original draft, Validation, Methodology, Formal analysis. **Elaheh Molaakbari:** Writing – review & editing, Writing – original draft, Software, Formal analysis, Data curation. **Mehdi Bamorovat:** Writing – review & editing, Writing – original draft, Validation, Software, Methodology, Formal analysis. **Zahra Babaei:** Writing – review & editing, Writing – original draft, Visualization, Methodology, Formal analysis. **Ebrahim Eskandari:** Writing – review & editing, Writing – original draft, Validation, Methodology, Formal analysis. **Ehsan Salarkia:** Writing – review & editing, Writing – original draft, Software, Formal analysis, Data curation. **Marzieh Asadi:** Writing – review & editing, Writing – original draft, Methodology, Formal analysis, Data curation.

## Declaration of competing interest

The authors declare that they have no known competing financial interests or personal relationships that could have appeared to influence the work reported in this paper.

## Data Availability

The datasets generated during and/or analyzed in the current study are available from the corresponding author upon reasonable request.
